# The Cholesterol Metabolite 25-Hydroxycholesterol Activates Estrogen Receptor α-Mediated Signaling in Cancer Cells and in Cardiomyocytes

**DOI:** 10.1371/journal.pone.0016631

**Published:** 2011-01-31

**Authors:** Rosamaria Lappano, Anna Grazia Recchia, Ernestina Marianna De Francesco, Tommaso Angelone, Maria Carmela Cerra, Didier Picard, Marcello Maggiolini

**Affiliations:** 1 Department of Pharmaco-Biology, University of Calabria, Rende, Italy; 2 Department of Cell Biology, University of Calabria, Rende, Italy; 3 Département de Biologie Cellulaire, Université de Genève, Genève, Switzerland; Ecole Normale Supérieure de Lyon, France

## Abstract

**Background:**

The hydroxylated derivatives of cholesterol, such as the oxysterols, play important roles in lipid metabolism. In particular, 25-hydroxycholesterol (25HC) has been implicated in a variety of metabolic events including cholesterol homeostasis and atherosclerosis. 25HC is detectable in human plasma after ingestion of a meal rich in oxysterols and following a dietary cholesterol challenge. In addition, the levels of oxysterols, including 25HC, have been found to be elevated in hypercholesterolemic serum.

**Methodology/Principal Findings:**

Here, we demonstrate that the estrogen receptor (ER) α mediates gene expression changes and growth responses induced by 25HC in breast and ovarian cancer cells. Moreover, 25HC exhibits the ERα-dependent ability like 17β-estradiol (E2) to inhibit the up-regulation of HIF-1α and connective tissue growth factor by hypoxic conditions in cardiomyocytes and rat heart preparations and to prevent the hypoxia-induced apoptosis.

**Conclusions/Significance:**

The estrogen action exerted by 25HC may be considered as an additional factor involved in the progression of breast and ovarian tumors. Moreover, the estrogen-like activity of 25HC elicited in the cardiovascular system may play a role against hypoxic environments.

## Introduction

The estrogen receptor (ER) α and β belong to the nuclear receptor superfamily of ligand-inducible transcription factors [1]. The binding of 17β-estradiol (E2) to ERs induces receptor homodimerization and interaction with specific estrogen responsive elements (EREs) located within the regulatory regions of target genes [2]. Two separate activation domains mediate the ER-dependent transcriptional activity: the activation function (AF)-1 located in the amino-terminus and the hormone-dependent AF-2 located in the ligand binding domain (LBD) [3]. The binding of ligands to ER induces the formation of an AF-2 hydrophobic pocket which regulates the recruitment of cofactors and importantly receptor pharmacology [4]–[5].

Previous results obtained in both cell culture and animal models have indicated that ERα plays a crucial role in mediating the effects of estrogen in mammary gland development and breast cancer progression [6]–[7]. Moreover, estrogen has been shown to prevent vascular dysfunction and injury in an ER-dependent manner [8]–[10], to reduce cardiomyocyte hypertrophy [11] and to decrease infarct size and myocyte apoptosis in animal models with coronary occlusion [12]. Diverse mechanisms are involved in the protective action exerted by E2 in cardiomyocytes. Oxidative stress and the subsequent generation of reactive oxygen species (ROS) are thought to trigger cardiomyocyte apoptosis [13], while the ability of E2-activated ER to counteract these redox intermediates is very likely a key factor of overall cardioprotection. The cell response to lowered oxygen environment implicates the hypoxia-inducible-factor-1 (HIF-1), which regulates the expression of genes like the matricellular protein named Connective Tissue Growth Factor (CTGF) belonging to the CCN family of growth regulators (*cyr61*, CTGF and *nov*) [14]. The levels of CTGF are up-regulated during wound repair [15], inflammation [16], fibrotic disorders [17], tumor growth [18]–[19] and angiogenesis [18], [20]. Accumulating evidence has also suggested that CTGF exerts a crucial role in cardiac fibrotic processes, indicating it as a possible biomarker and a potential candidate for therapeutic intervention [21].

Oxysterols are hydroxylated derivatives of cholesterol that play important functions in lipid metabolism [22]. 25-hydroxycholesterol (25HC) which is synthesized from cholesterol by a specific hydroxylase [23], acts as a potent inhibitor of cholesterol biosynthesis in different cell types [24]. 25HC was detected in rat plasma after ingestion of a meal rich in oxysterols and following a dietary cholesterol challenge [25]. Likewise, the levels of oxysterols, including 25HC, were higher in hypercholesterolemic serum compared to those found in normocholesterolemic serum [26]. Next, mice deficient in the oxysterol-catabolizing enzyme oxysterol 7α-hydroxylase (Cyp7b) showed elevated concentrations of both 25HC and 27HC, suggesting a regulatory role elicited by Cyp7b on these hydroxylated cholesterol derivatives [27].

In the present study, we demonstrate that 25HC elicits estrogenic effects activating ERα-mediated signaling either in breast and ovarian cancer cells or in cardiomyocytes. The results obtained were confirmed, at least in part, in isolated and perfused rat hearts.

## Materials and Methods

### Reagents

17β-estradiol (E2), 25-hydroxycholesterol (25HC), cobalt chloride (CoCl_2_), PD98059 (PD), SB202190 (SB) and actinomycin D were purchased from Sigma-Aldrich, Inc., Milan, Italy. The experiments performed using 25HC from Sigma-Aldrich, Inc., Milan (Italy) were confirmed with 25HC purchased from Research Plus, Inc., Barnegat, NJ. ICI 182,780 (ICI) was obtained from Tocris Chemicals. All compounds were solubilized in dimethyl sulfoxide (DMSO), except E2 and PD, which were dissolved in ethanol.

### Cell Culture

Breast cancer MCF7 and human embryonal kidney Hek293 cells were maintained in DMEM with phenol red supplemented with 10% FBS. Breast SkBr3 and ovarian BG-1 cancer cells were maintained in RPMI1640 and DMEM, respectively, without phenol red supplemented with 10% FBS. The murine cardiomyocyte-like cell line HL-1 was kindly provided by Dr. William C. Claycomb (Louisiana State University Medical Center, New Orleans, LA). HL-1 cells were cultured according to the published protocol [28] in Claycomb medium (JRH Biosciences, Sigma-Aldrich, Inc., Milan, Italy) supplemented with 10% FBS (JRH Bioscience, Sigma-Aldrich, Inc., Milan, Italy), 100µg/ml penicillin/streptomycin, 0.1mM norepinephrine (Sigma-Aldrich, Inc., Milan, Italy) and 2mM *l*-glutamine (Invitrogen, Milan, Italy). All cell lines were grown in a 37°C incubator with 5% CO_2_. For hypoxic stimulation HL-1 cells were treated with CoCl_2_ or cultured in presence of low oxygen tension (2% O_2_) in a HeraCell incubator (ThermoScientific-Heraeus, Milan, Italy). All cell lines to be processed for immunoblot and RT-PCR assays were switched to medium without serum and phenol red 24 h before treatments.

### Transfections, luciferase assays and gene silencing experiments

Plasmids and Luciferase Assays were previously described [29]–[32]. In particular, we used expression vectors encoding the full length ERα and the ERβ form encoding the 485 aa protein [29]–[32]. Cells were plated into 10-cm dishes, maintained in serum-free medium for 24 h and then transfected for additional 24 h before treatments using Fugene6 (Roche Molecular Biochemicals, Milan, Italy) and appropriate control vectors. The SureSilencing™ shRNA plasmids for mouse HIF-1α and respective negative control plasmids (shRNA) were purchased from Superarray Bioscience Corporation (Frederick, MD, USA) and were used according to the manufacturer's recommendations (Cat. KM03799P). Briefly, cells were plated into 10-cm dishes, and then transfected in serum-free medium for 24h before treatments with a mixture containing 15µl/plate Fugene6 Reagent and 5µg/plate control shRNA or shRNA HIF-1α (shHIF-1α) plasmid. For HIF-1α silencing, the manufacturer provides 4 separate short hairpin RNA (shRNA) pre-designed sequences for the same gene, packaged in 4 separate plasmid backbones (numbered as 1–4). In our experiments, a single independent shRNA plasmid sequence (n.3) was sufficient to silence HIF-1α gene expression. The control shRNA is a scrambled artificial sequence which does not match any human, mouse or rat gene.

### ERα binding assays

The ability of 25HC to compete with [3H]E2 for binding to ERα was evaluated either in MCF7 cells or using Hek293 cell lysates in presence or absence of two picomoles of purified recombinant human ERα protein purchased from PanVera, Invitrogen S.r.l. Milan, Italy. MCF7 cells were stripped of any estrogen by keeping them in medium without serum for 2 days, thereafter cells were incubated with 1nM [2,4,6,7-3H]E2 (89 Ci/mmol; Ge Healthcare, Milan, Italy) and increasing concentrations of nonlabeled E2 or 25HC for 2 h at 37°C in a humidified atmosphere of 95% air/5% CO2. After removal of the medium, cells were washed with ice-cold PBS/0.1% methylcellulose twice, harvested by scraping and centrifugation, and lysed with 100% ethanol, 500 µl per 60 mm dish, for 10 min at room temperature [33]. The radioactivity of extracts was measured by liquid scintillation counting. Binding assay was also performed using Hek293 whole cell lysates. Cells were stripped of any estrogen by keeping them in medium without serum for 2 days, and then lysed in 500µl of RIPA buffer (20mM Tris-HCl, pH 7.5; 100mM NaCl; 0.5% Nonidet P-40; 0.5mM EDTA) in presence of a mixture of protease inhibitors containing 1,7 mg/ml aprotinin, 1 mg/ml leupeptin, 200 mmol/liter phenylmethylsulfonylfluoride, 200 mmol/liter sodiumorthovanadate and 100 mmol/liter sodium fluoride. Protein concentration was determined using Bradford reagent according to the manufacturer's recommendations (Sigma-Aldrich, Inc., Milan, Italy). Equal amounts of whole-protein extract were incubated in the absence or presence of two picomoles of recombinant ERα and incubated with 1nM [3H]E2 and increasing concentrations of unlabeled E2 or 25HC for 2 h at 4°C. Bound and free radioligands were separated on Sephadex G-25 PD-10 columns. The amount of receptor-bound [3H]E2 was determined by liquid scintillation counting.

### Chromatin immunoprecipitation (ChIP) and Re-ChIP assays

MCF7 cells were grown in 10-cm dishes to 70–80% confluence, shifted to serum free medium for 24 h and then treated with vehicle, 10nM E2 or 1µM 25HC for 1 h. Thereafter, cells were cross-linked with 1% formaldehyde and sonicated. Supernatants were immunocleared with sonicated salmon DNA/protein A agarose (Upstate Biotechnology, Inc., Lake Placid, NY) and immunoprecipitated with anti-ERα antibody or non specific IgG (Santa Cruz Biotechnology, DBA, Milan, Italy). Pellets were washed, eluted with a buffer consisting of 1% SDS and 0.1 mol/L NaHCO3, and digested with proteinase K. DNA was obtained by phenol/chloroform extraction and precipitated with ethanol. A 4 µl volume of each sample was used as template to amplify an ERE-containing region located in the pS2 promoter by real-time PCR (Applied Biosystems, Milan, Italy). The primers used were 5′-GGCCATCTCTCACTATGAATCACTTCTGC-3′ (pS2 forward) and 5′-GGCAGGCTCTGTTTGCTTAAAGAGCG-3′ (pS2 reverse). Data were normalized to the input for the immunoprecipitation. In Re-ChIP experiments, complexes were eluted by incubation for 30 min in Re-IP buffer (0.5 mM dithiothreitol, 1% Triton X-100, 2 mM EDTA, 20 mM Tris-Cl pH 8.1, 150 mM NaCl) and subjected to the ChIP procedure, using anti-SRC-1, SRC-3 and CBP antibodies (all purchased from Santa Cruz Biotechnology, DBA, Milan, Italy).

### Reverse transcription and real-time PCR

Gene expression was evaluated by real-time PCR as we previously described [32]. For pS2, PR, Cathepsin D, Cyclin A, Cyclin D1, HIF-1α, CTGF, FAS, SERPINF1, HSPA1L, SELENBP1 and the ribosomal protein 18S, which was used as a control gene to obtain normalized values, the primers were: 5′-GCCCCCCGTGAAAGAC-3′ (pS2 forward) and 5′-CGTCGAAACAGCAGCCCTTA-3′ (pS2 reverse); 5′-GAGTTGTGAGAGCACTGGATGCT-3′ (PR forward) and 5′-CAACTGTATGTCTTGACCTGGTGAA-3′ (PR reverse); 5′-CTGGATCCACCACAAGTACAACA-3′ (Cathepsin D forward) and 5′-CGAGCCATAGTGGATGTCAAAC-3′ (Cathepsin D reverse); 5′-TGCACCCCTTAAGGATCTTCCT-3′ (Cyclin A forward) and 5′-GTGAACGCAGGCTGTTTACTGT-3′ (Cyclin A reverse); 5′-GTCTGTGCATTTCTGGTTGCA-3′ (Cyclin D1 forward) and 5′-GCTGGAAACATGCCGGTTA-3′ (Cyclin D1 reverse); 5′-TGCATCTCCATCTTCTACCCAAGT-3′ (HIF-1α forward) and 5′-CCGACTGTGAGTGCCACTGT-3′ (HIF-1α reverse); 5′-CATTAAGAAGGGCAAAAAGTGCAT-3′ (CTGF forward) and 5′-TGCAGCCAGAAAGCTCAAACT-3′ (CTGF reverse); 5′-CGCTCGGCATGGCTATCT-3′ (FAS forward) and 5′-CTCGTTGAAGAACGCATCCA-3′ (FAS reverse); 5′-CCCGGATCGTCTTTGAGAAG-3′ (SERPINF1 forward) and 5′-TCCAGAGGTGCCACAAAGCT-3′ (SERPINF1 reverse); 5′-CCGTGCCAGCCTATTTCAAT -3′ (HSPA1L forward) and 5′- AGCAATCACACCTGCATCCTT-3′ (HSPA1L reverse); 5′-GCAGCGCCATGAGATTGTG-3′ (SELENBP1 forward) and 5′-CGGATCTCCAAGGGAATAAGC-3′ (SELENBP1 reverse), and 5′-GGCGTCCCCCAACTTCTTA-3′ (18S forward) and 5′-GGGCATCACAGACCTGTTATT-3′ (18S reverse), respectively.

### Immunoblotting

Cell lysates and immunoblotting assays were performed as previously described [32]. The antibodies against ERα (F-10), Cyclin D1 (M-20), CTGF (L-20), phosphorylated ERK1/2 (E-4) and ERK2 (C-14), β-actin (C-2) and β-tubulin (H-235-2) were purchased from Santa Cruz Biotechnology (DBA, Milan, Italy). ERβ and HIF-1α were purchased from R&D Systems, Inc. (Milan, Italy). Phospho-p38MAPK (Thr180/Tyr182) and p38MAPK were purchased from Cell Signalling Technology, Inc. (Milan, Italy).

### Proliferation and TUNEL assays

Quantitative proliferation assays were performed as previously described [32]. HL-1 cells were plated in 2-well Lab-Tek® II chamber slides and treated for 18 h. After removing medium, cells were fixed in 4% buffered paraformaldehyde (pH 7.4) for 30 min. Slides were rinsed twice in PBS, pH 7.4. An *in situ* cell death detection kit (DeadEnd™, Fluorometric TUNEL System, Promega Corp, Milan, Italy) was subsequently used to perform DNA 3′-hydroxyl end labelling by TUNEL (Terminal deoxynucleotidyl Transferase (TdT)-mediated dUTP-X Nick End Labelling) assay as previously described [34]. Labelling of cells was shown to be correlated with typical morphological criteria of apoptosis. DNA was labelled in 3′-ends with fluorescein-dUTP by incubation at 37°C with reaction buffer containing TdT in a humidified chamber for 1 h. Nuclei of cells were stained with propidium iodide. After three washes in PBS, apoptotic DNA fragmentation was directly detected by visualization of labelled DNA using fluorescent microscope. For negative control, slides were incubated without TdT; to obtain positive control, slides were treated with 1µg DNAse I/ml for 10 min at room temperature before exposure to fluorescein dUTP and TdT.

### Isolated and perfused heart preparations

All experimental protocols were approved by the Committee on the Use of Animals of the Pharmaco-Biology Department at the University of Calabria (approval ID 110/2000A). The procedures followed in the study were in accordance with the European Community standards on the care and use of laboratory animals and with the *Guide for the Care and Use of Laboratory Animals* published by the US National Institutes of Health (NIH Publication No. 85-23, revised 1996). Adult male rats (Wistar, 220–280g, Harlan, Udine, Italy) were anaesthetized by i.p. injection of ethyl carbamate (2 g/kg body weight). Hearts were then dissected out and connected to a Langendorff apparatus for perfusion with a Krebs–Henseleit solution (KHs) composed of (in mM) NaCl 113, KCl 4.7, NaHCO3 25, MgSO4 1.2, CaCl2 1.8, KH2PO4 1.2, glucose 11, mannitol 1.1, Na-pyruvate 5 and gassed with 95%O_2_-5%CO_2_ (pH7.4, 37°C). KHs was delivered at a constant flow-rate of 12 mL/min. To measure cardiac activity, a water-filled latex balloon, connected to a BLPR gauge (WRI, Inc. USA), was inserted through the mitral valve into the left ventricle to allow isovolumic contractions and to continuously record mechanical parameters. The balloon was progressively filled with water to obtain an initial left ventricular end diastolic pressure of 5 to 8 mmHg [35]. All hearts were perfused for a 15 min equilibration period. After the equilibration period, the hearts (n = 3) were randomly assigned to one of the following groups: Group 1 (control): KH gassed with 95%O_2_-5%CO_2_; Group 2: KH gassed with 95%O_2_-5%CO_2_ plus 1nM E2; Group 3: KH gassed with 95%O_2_-5%CO_2_ plus 100nM 25HC; Group 4: KH gassed with 50%O_2_-45%N_2_-5%CO_2_; Group 5: KH gassed with 50%O_2_-45% N_2_-5%CO_2_ plus 1nM E2; Group 6: KH gassed with 50%O_2_-45% N_2_-5%CO_2_ plus 100nM 25HC.

All hearts were perfused for 60 minutes to investigate the effect of each compound and of the different oxygen levels on the changes of CTGF and HIF-1α mRNA expression. Solution containing treatments was freshly prepared before experiments. Left ventricular pressure, heart rate and coronary flow were monitored throughout the perfusion protocol. At the end of the perfusions, ventricles were excised and immediately processed for RNA extraction.

### Statistical Analysis

Statistical analysis was done using ANOVA followed by Newman-Keuls' testing to determine differences in means. P<0.05 was considered as statistically significant.

## Results

### 25HC activates ERα in cancer cells

**Figure 1:**
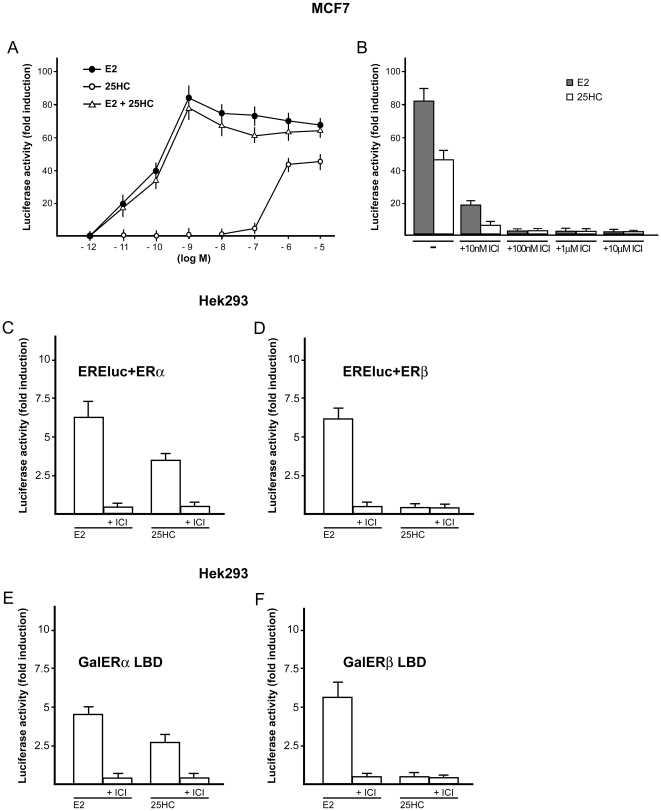
25HC activates ERα. (A) MCF7 cells were transfected with an ER luciferase reporter gene along with the internal transfection control Renilla Luciferase and treated with increasing concentrations (logarithmic scale) of E2 and 25HC. Moreover, cells were treated simultaneously with similar doses of each compound. The normalized luciferase activity values of cells treated with vehicle (-) were set as 1-fold induction, upon which the activity induced by treatments was calculated. (B) MCF7 cells transfected with the ER reporter gene were treated with 10nM E2 or 1µM 25HC alone and in combination with increasing concentration of the ER antagonist ICI, as indicated. Each data point represents the mean ± SD of three experiments performed in triplicate. (C–F) Hek293 cells were transfected with ER luciferase reporter gene (EREluc) and ERα (C) or ERβ (D) expression plasmids, with Gal4 reporter gene GK1 and the Gal4 fusion proteins encoding the Ligand Binding Domain (LBD) of ERα (GalERα) (E) or ERβ (GalERβ) (F) and treated with 10nM E2 or 1µM 25HC alone and in combination with 10µM ER antagonist ICI, as indicated. Each data point represents the mean ± SD of three experiments performed in triplicate.

We began evaluating whether 25HC is able to transactivate a transiently transfected ER reporter gene in breast cancer cells (MCF7), which express ERα and not ERβ as judged by RT-PCR (data not shown). Interestingly, 25HC activated ERα in a dose-dependent manner, although with a lower efficacy compared to E2 (Fig. 1A). We next evaluated whether 25HC could antagonize the transcriptional activation induced by E2. MCF7 cells were then treated simultaneously with increasing concentrations of both 25HC and E2, however the transcriptional responses were similar to those obtained used E2 alone (Fig. 1A). On the contrary, the luciferase activity induced by 10nM E2 or 1µM 25HC were abrogated using increasing doses of the ER antagonist ICI (Fig. 1B), suggesting that ERα mediates the transcriptional activation upon exposure to each compound. To provide further data regarding the ability of 25HC to activate ERα and to evaluate whether ERβ could also respond to 25HC, we transiently transfected ER-negative Hek293 cells with the ER reporter gene along with the expression vector encoding ERα or ERβ. Of note, only ERα expression allowed 1µM 25HC to induce the luciferase activity, which was abolished by 10µM ICI (Fig. 1C–D). To provide further evidence regarding the ability of 25HC to activate ERα but not ERβ, we turned to a completely heterologous system. In Hek293 cells, 1µM 25HC activated a chimeric protein consisting of the DNA binding domain (DBD) of the yeast transcription factor Gal4 and the LBD of ERα but not ERβ (Fig. 1E–F). Again, the transactivation of ERα was abolished by 10µM ICI (Fig. 1E–F). To corroborate the aforementioned findings, we ascertained whether 25HC could induce the expression of the ERβ target genes SERPINF1, HSPA1L, SELENBP1, which were reported to respond to E2 treatment [36]. Using Hek293 cells transiently transfected with an ERβ expression plasmid, the transcription of these genes was stimulated by E2 but not by 25HC (Fig. S1). Taken together, these results demonstrate that 25HC activates ERα in a selective manner and show that ERα-LBD is sufficient for the transcriptional response.

**Figure 2:**
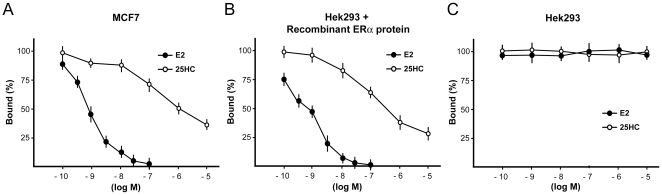
ERα ligand binding assays. The graphs show residual binding of the radioactive tracer in the presence of increasing concentrations of unlabelled E2 and 25HC in MCF7 cells (A), in Hek293 cell lysates in the presence (B) or absence of recombinant ERα protein (C). Each data point represents the mean ± SD of triplicate samples of three separate experiments. Note that the amount of tracer bound in the absence of competitor was arbitrarily set to 100% and that the underlying absolute values differ between the three panels.

On the basis of the results obtained in transfection experiments, we asked whether 25HC could behave as a ligand and compete with E2 for the binding to ERα. In a whole cell binding assay with MCF7 cells 25HC displaced the radiolabeled E2 in a dose-dependent manner (Fig. 2A). Similarly, when recombinant ERα was added to a Hek293 cell lysate, which by itself does not bind E2, 25HC competed efficiently against the tracer E2 (Fig. 2B–C). The results of these binding assays are compatible with the idea that 25HC binds ERα directly, but further experiments will be necessary to establish the mode of action unambiguously.

### 25HC induces the recruitment of ERα to the pS2 promoter sequence in MCF7 cells

**Figure 3:**
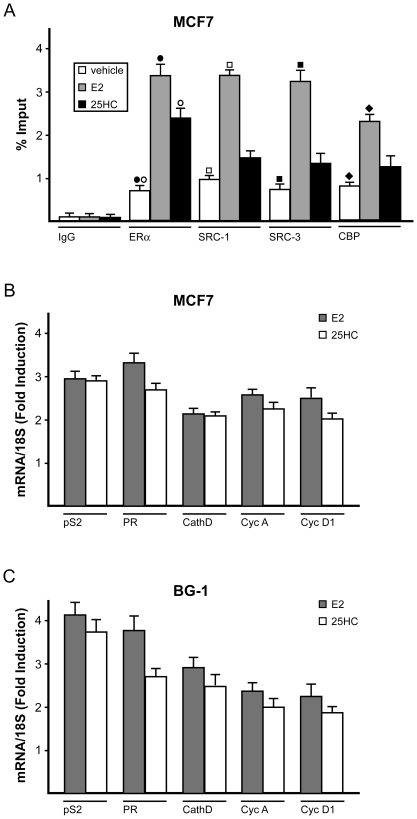
25HC up-regulates the expression of estrogen target genes. (A) E2 and 25HC induce the recruitment of ERα to the ERE site located in the pS2 promoter sequence in MCF7 cells. Cells were treated for 1 h with vehicle, E2 (10nM) or 25HC (1µM) and submitted to the chromatin immunoprecipitation procedure using anti-ERα or nonspecific anti-IgG antibodies. For Re-ChIP assays, anti-SRC-1, SRC-3 and CBP antibodies were used. The amplified sequences were evaluated by real-time PCR. (B–C) evaluation of mRNA expression of pS2, Progesterone Receptor (PR), Cathepsin D, Cyclin A and Cyclin D1 by real-time PCR in MCF7 and BG-1 cells. Cells were treated for 24h with 10nM E2 and 1µM 25HC. Results obtained from experiments performed in triplicate were normalized for 18S expression and shown as fold change of RNA expression compared to cells treated with vehicle. Each data point represents the mean ± SD of three experiments performed in triplicate. (•), (°), (▪), (□) (♦) indicate *p*<0.05 for cells receiving vehicle (–) versus treatments.

On the basis of the aforementioned findings, we evaluated whether 25HC could induce the recruitment of ERα to targeted ERE-containing DNA region within the pS2 promoter sequence. Performing a chromatin immunoprecipitation (ChIP) assay in MCF7 cells, 1h treatment with 1µM 25HC induced the recruitment of ERα to the pS2 promoter as observed using 10nM E2 (Fig. 3A). Moreover, to verify whether 25HC may recruit co-activators to the pS2 promoter, we performed Re-ChIP assay using antibodies against SRC-1 and SRC-3, which belong to the steroid receptor co-activator (SRC) families and against CBP (CREB-binding protein) [37]. All co-activators were efficiently recruited to the pS2 promoter exposing MCF7 cells to 10nM E2, however 1µM 25HC showed a slight efficacy (Fig. 3A). Hence, E2 and 25HC display a different ability in recruiting co-activators, which contribute to the agonistic potency of ligands.

### 25HC regulates the expression of ERα target genes in MCF7 and BG-1 cells

**Figure 4:**
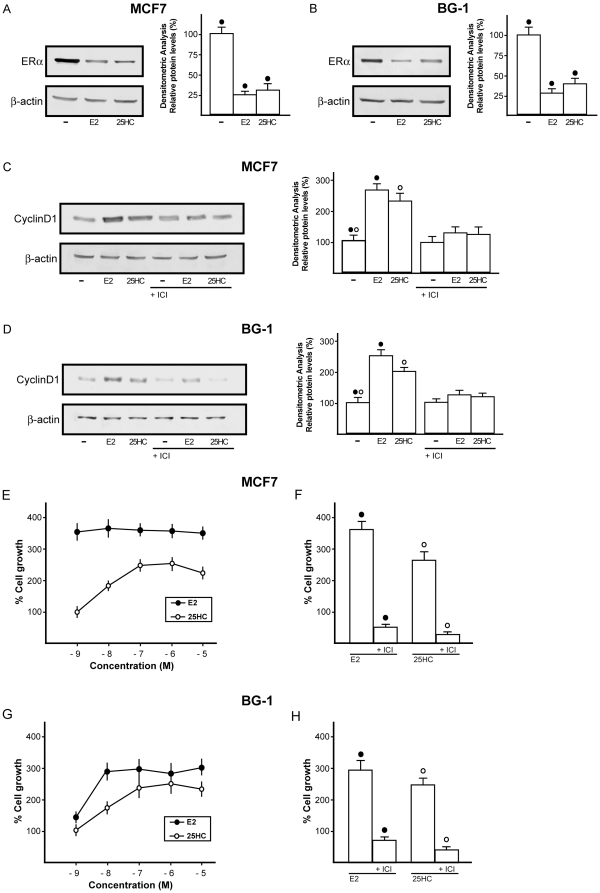
25HC induces cancer cell proliferation. Immunoblots of ERα (A, B) and Cyclin D1 (C, D) from MCF7 and BG-1 cells. Cells were treated for 24h with vehicle (-), 10nM E2 or 1µM 25HC and in presence of 10µM ER-antagonist ICI, as indicated. β-actin serves as loading control. 25HC induces proliferative effects in MCF7 and BG-1 cells (E–H). Cells were treated for 5 days with increasing concentrations (logarithmic scale) of E2 and 25HC and counted on day 6 (E,G). Cells were treated with 10nM E2 or 1µM 25HC and in combination with 10µM ER-antagonist ICI and counted on day 6 (F,H). Proliferation of cells receiving vehicle was set as 100% upon which cell growth induced by treatments was calculated. Each data point is the average ± SD of three independent experiments. (•), (°) indicate *p*<0.05 for cells receiving vehicle (–) versus treatments.

We next evaluated the potential of 25HC to regulate the expression of well known ERα target genes, such as pS2, PR, Cathepsin D, Cyclin A and Cyclin D1 in MCF7 breast and BG-1 ovarian cancer cells. As determined by real-time RT-PCR, a 24 h exposure to 1µM 25HC up-regulated the mRNA expression of all genes examined, similar to the treatment with 10nM E2 (Fig. 3B–C). To further corroborate these findings, we investigated the ability of 25HC to modulate the expression of ERα and Cyclin D1 protein levels in MCF7 and BG-1 tumor cells. To date, the down-regulation of ERα by estrogen has been considered a hallmark of receptor activation [38], while the up-regulation of Cyclin D1 by E2 has been extensively reported [39]. Notably, a 24 h exposure to 1µM 25HC down-regulated ERα protein levels (Fig. 4A–B). On the contrary, a 24 h treatment with 1µM 25HC up-regulated Cyclin D1 protein expression, which was abrogated by 10µM ICI (Fig. 4C–D). Altogether, these results suggest that 25HC is able to regulate the expression of ERα target genes like E2.

### 25HC induces proliferative effects in MCF7 and BG-1 cells

Then, we sought to evaluate the biological counterpart of the above-mentioned effects exerted by 25HC. Growth assays performed in MCF7 and BG-1 cancer cells demonstrated that 25HC is able to induce proliferative effects in a dose dependent manner (Fig. 4E and 4G). Next, the growth responses to 10nM E2 and 1µM 25HC were abolished by 10µM ICI (Fig. 4F and 4H), suggesting that ERα mediates cell proliferation induced upon exposure to both compounds.

### 25HC mimics the effects of E2 against hypoxia-induced apoptosis in cardiomyocytes

**Figure 5:**
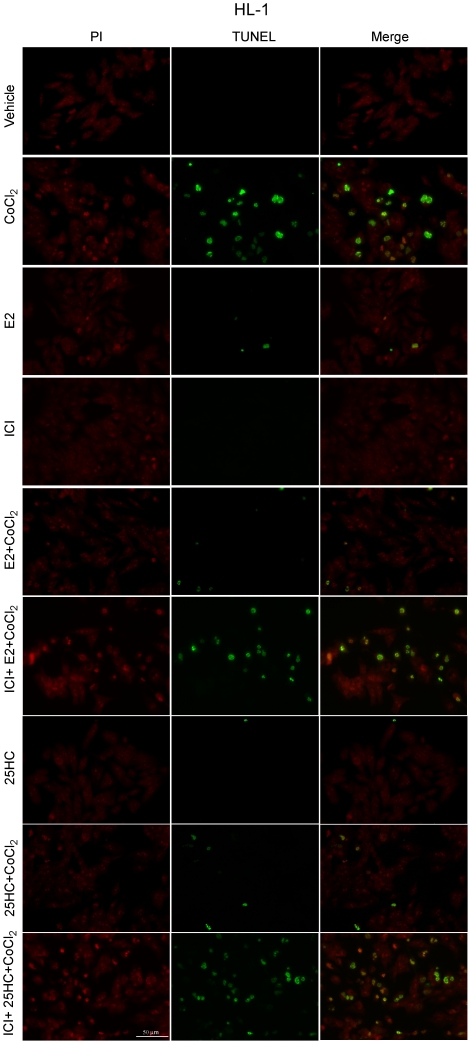
E2 and 25HC prevent CoCl2-induced apoptosis in HL-1 cells. Apoptotic changes were detected using TUNEL (in green) and nuclei were stained by propidium iodide (PI, in red), as indicated. Representative photographs after 18h treatment with vehicle and 100µM CoCl2, 10nM E2, 1µM 25HC alone, or a combination of 100µM CoCl2 with 10nM E2 in presence or absence of 10µM ICI, 100µM CoCl2 with 1µM 25HC in presence or absence of 10µM ICI.

To further evaluate the estrogenic properties of 25HC, we turned to a completely different model system like the HL-1 cardiomyocytes [28], which express ERα and very low ERβ levels as judged by RT-PCR (data not shown). It has been reported that E2 protects cells from hypoxic insult in different cell contexts [40]–[42]. Hence, we evaluated whether E2 and 25HC could protect HL-1 cardiomyocytes from apoptosis induced by the well known hypoxia-mimetic agent CoCl_2_ [43]–[44]. Determining DNA degradation by TUNEL assay, approximately 70% of HL-1 cells resulted positive for TUNEL staining after exposure to CoCl_2_ (Fig. 5 and Fig. S2). Of note, the percentage of apoptotic cells was significantly reduced in presence of either 10nM E2 or 1µM 25HC (Fig. 5 and Fig. S2). The protective effects exerted by both compounds were abrogated using 10µM ICI, which alone did not induce apoptosis (Fig. 5 and Fig. S2). Taken together, the results shown indicate that E2 and 25HC may protect cardiomyocytes from hypoxia-induced apoptosis in an ER-dependent manner.

### E2 and 25HC prevent the hypoxia-induced expression of HIF-1α and CTGF in cardiomyocytes

**Figure 6:**
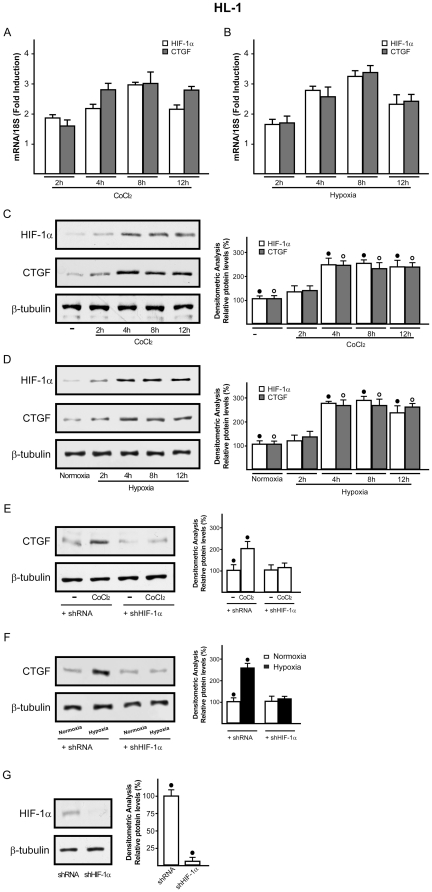
Evaluation of HIF-1α and CTGF expression in HL-1 cells. HIF-1α and CTGF mRNA expression were evaluated by real-time PCR after 100µM CoCl_2_ treatment (A) and incubating HL-1 cells under hypoxia (2%O_2_) (B). Results obtained from experiments performed in triplicate were normalized for 18S expression and shown as fold change of RNA expression compared to cells treated with vehicle (A) or cultured under normoxia (B). (C) immunoblots of HIF-1α and CTGF from HL-1 cells after treatment with vehicle (-) or 100µM CoCl_2_, as indicated. β-tubulin serves as a loading control. Side panel shows densitometric analysis of the blots, normalized to β-tubulin. (D) immunoblots of HIF-1α and CTGF from HL-1 cells cultured under normoxia or in presence of low oxygen tension (2% O_2_), as indicated. β-tubulin serves as a loading control. Side panel shows densitometric analysis of the blots, normalized to β-tubulin. Cells were transfected with a control shRNA or shHIF-1α and exposed for 8h to 100µM CoCl_2_ (E) or hypoxic conditions (2% O_2_) (F). Side panels show densitometric analysis of the blots, normalized to β-tubulin. (G) efficacy of HIF-1α silencing obtained using shHIF-1α. Each data point is the average ± SD of three independent experiments. (•), (°) indicate *p*<0.05 for cells receiving vehicle (–) versus treatments or for cells cultured under normoxia versus cells exposed to hypoxia.

**Figure 7:**
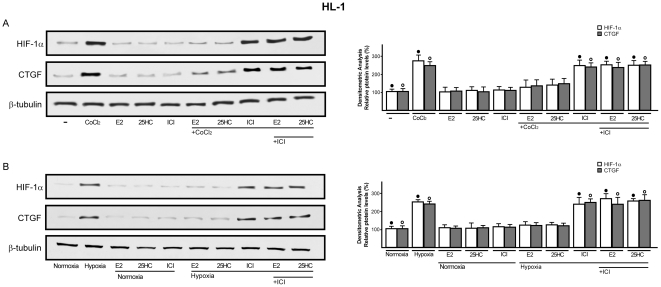
Immunoblots of HIF-1α and CTGF from HL-1 cells. (A) cells were treated for 8h with 100µM CoCl_2_ or (B) exposed to hypoxia (2% O_2_), in combination with 10nM E2, 1µM 25HC and 10µM ICI, as indicated. β-tubulin serves as loading control. Data (mean ± SD) are representative of three independent experiments. Side panels show densitometric analysis of the blots, normalized to β-tubulin. (•), (°) indicate *p*<0.05 for cells receiving vehicle (–) versus treatments or for cells cultured under normoxia versus cells exposed to hypoxia.

**Figure 8:**
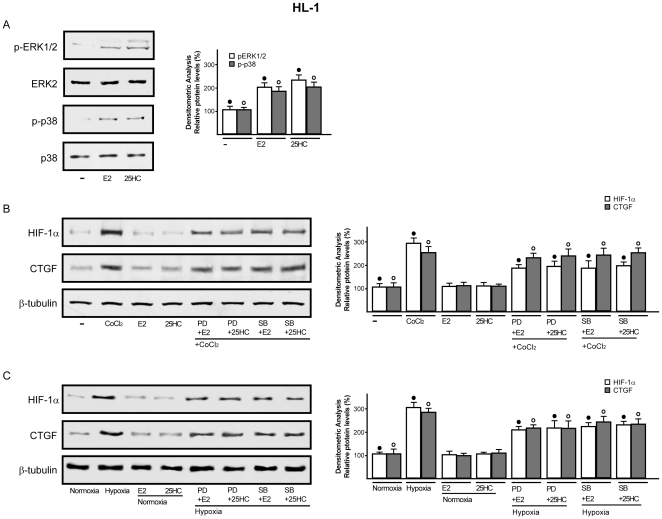
25HC prevents the hypoxia-induced expression of HIF-1α and CTGF through kinase-mediated signalling. (A) immunoblots of p-ERK1/2 and p-p38 from HL-1 cells treated for 10 min with vehicle (-), 10nM E2 and 1µM 25HC. Total ERK/2 and p38 serve as loading control. (B) immunoblots of HIF-1α and CTGF from HL-1 cells exposed for 8h to 100µM CoCl_2_ or hypoxia (2% O_2_) (C), in combination with 10nM E2, 1µM 25HC and 10µM PD or 10µM SB, as indicated. β-tubulin serves as loading control. Data (mean ± SD) are representative of three independent experiments. Side panels show densitometric analysis of the blots, normalized to β-tubulin. (•), (°) indicate *p*<0.05 for cells receiving vehicle (–) versus treatments or for cells cultured under normoxia versus cells exposed to hypoxia.

It has been previously reported that HIF-1α mediates CTGF expression stimulated by hypoxia in different cell contexts [45]–[47]. Whilst the potential regulation of HIF-1α by E2 has been suggested in certain models [48]–[49], the estrogen ability to influence hypoxia-induced CTGF expression in the cardiovascular system remains to be investigated. On the basis of these observations, we ascertained that HIF-1α and CTGF are up-regulated at both mRNA and protein levels in a time-dependent manner by 100µM CoCl_2_ in HL-1 cells (Fig. 6A,C). Similar responses were observed incubating HL-1 cells in presence of low oxygen tension (2% O_2_) (Fig. 6B,D). Next, the hypoxia-induced expression of HIF-1α and CTGF was abolished using the inhibitor of DNA-primed RNA synthesis actinomycin D (Fig. S3), suggesting that transcriptional mechanisms are responsible for the up-regulation of both HIF-1α and CTGF. Then, we determined that the silencing of HIF-1α abrogates CTGF induction observed either upon exposure to CoCl_2_ or hypoxic conditions (2% O_2_) (Fig. 6E–F), indicating that HIF-1α mediates the up-regulation of CTGF by hypoxia in HL-1 cells. Thereafter, we asked whether E2 and 25HC might influence HIF-1α and CTGF expression. Interestingly, we found that either 10nM E2 or 1µM 25HC prevent the up-regulation of HIF-1α and CTGF either by CoCl_2_ or low oxygen tension (2% O_2_) at both mRNA (Fig. S4) and protein levels (Fig. 7A–B). However, this ability of E2 and 25HC was abolished using 10µM ICI (Fig. 7A–B and Fig. S4), suggesting that the action of both compounds involves an ER-mediated mechanism. To provide further data regarding the intracellular signaling regulating HIF-1α and CTGF expression, we established that 10nM E2 and 1µM 25HC phosphorylate ERK1/2 and p38MAPK in a rapid manner (Fig. 8A). Accordingly, in presence of the inhibitors of these kinases, PD and SB respectively, both E2 and 25HC lost the repressive effects exerted on the up-regulation of HIF-1α and CTGF at both mRNA and protein levels (Fig. 8B–C and Fig. S4). Altogether, these results suggest that 25HC triggers both ER and kinase-mediated signaling similar to E2.

### HIF-1α and CTGF mRNA induction by hypoxia is prevented by E2 and 25HC in perfused rat hearts

**Figure 9:**
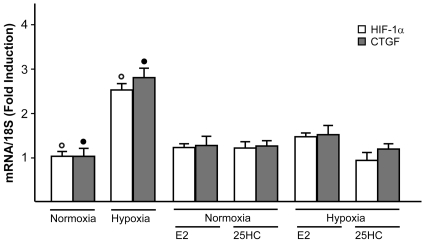
Evaluation of HIF-1α and CTGF mRNA expression in hypoxic rat heart preparations. HIF-1α and CTGF mRNA expression was evaluated by real time PCR after 1h exposure to low pO2 levels (40%) and in presence of 1nM E2 or 100nM 25HC treatment, as indicated. mRNA values obtained from control and hypoxic rat hearts were normalized for 18S expression and shown as fold change in triplicate experiments. (°), (•) indicate *p*<0.05 for control versus hypoxic rat hearts.

On the basis of the aforementioned results, we examined whether hypoxia could increase HIF-1α and CTGF mRNA expression in isolated and perfused rat heart preparations. Following the experimental conditions described in Materials and Methods section, rat ventricles were excised after 1h exposure to low pO2 levels (40%) and subjected to RNA extraction and real-time PCR evaluation. Interestingly, the up-regulation of both HIF-1α and CTGF expression by hypoxia was abrogated in presence of 1nM E2 or 100nM 25HC (Fig. 9), confirming the results obtained in HL-1 cells.

## Discussion

25HC has been implicated in a variety of relevant metabolic-related events, such as cellular cholesterol homeostasis and atherosclerosis [22]. Moreover, 25HC was shown to be more potent than cholesterol in stimulating cells to respond to high sterol levels either decreasing 3-hydroxy-3-methylglutaryl coenzyme A (HMG CoA) reductase activity or by increasing cholesterol esterification, thereby leading to a decreased synthesis and/or augmented storage of cholesterol [50]. In the cell, 25HC is synthesized from cholesterol by auto-oxidation or enzymatically by a non-heme, iron-containing protein [25], [51]. Significant levels of 25HC are produced in different cell contexts [25], [52]–[53] and even in a selective manner with respect to other cholesterol derivatives, as occurs in astrocytes [54]. Several studies in animals and humans have shown that oxysterols, including 25HC, are absorbed in the gut, transported into the circulation within chylomicrons and then are taken up into tissues depending on diet and metabolism [55].

Moreover, previous investigations reported different 25HC plasma levels, ranging from ng/ml [56] to µg/ml [57]. In addition, 25HC has been demonstrated to promote the differentiation of Leydig cells [58], hepatocytes and keratinocytes [59]–[60] and to induce fatty acid synthase (FAS) expression [61], as we confirmed to occur also in a cell context lacking ERs like SkBr3 breast cancer cells (Fig. S5). Likewise, 25HC stimulated interleukin-8 secretion in monocytic THP-1 cells activating the ERK/c-fos/AP-1 transduction pathway in a calcium-dependent manner [62] and exerted dose-dependent effects on cell proliferation [54], [63]–[64].

In this study, we have demonstrated that 25HC is able 1) to activate ERα, possibly by binding directly as an agonistic ligand, 2) to up-regulate diverse estrogen target genes, 3) to modulate ERα and Cyclin D1 protein levels and 4) to stimulate growth effects in breast and ovarian cancer cells. In addition, we have shown that 25HC in cardiomyocytes elicits an ER-mediated prevention of hypoxia-dependent apoptosis and inhibition of the up-regulation of hypoxia-induced expression of HIF-1α and CTGF. Noteworthy, 25HC mimicked the action of E2 to prevent hypoxia-induced HIF-1α and CTGF expression in isolated and perfused rat heart preparations.

Recently, it has been reported that the 27HC derivative can interact with and regulate the activity of both ER isoforms, triggering diverse biological actions either in vitro or in vivo [65]. In particular, 27HC acted as a selective estrogen receptor modulator (SERM) on the basis of its agonist/antagonist activities elicited in a cell- and promoter-dependent manner [66]–[67]. In breast cancer cells 27HC stimulated both gene transcription and growth effects, suggesting its potential role in the progression of breast tumor in obese/hypercholesterolemic women [66]. Interestingly, 27HC inhibited the transcription-dependent and even transcription-independent estrogen-mediated production of nitric oxide by vascular cells leading to a decreased vasorelaxation of rat aorta upon estrogen exposure [67]. Consequently, 27HC was indicated as a contributing factor to the loss of estrogen protection in vascular disease. Collectively, in estrogen-dependent tumor cells 27HC and 25HC exhibited similar stimulatory action, however they displayed distinct ER-mediated functions according to the cardiovascular model system used.

In breast and ovarian cancer, E2 stimulates proliferative effects through ERα [68]–[70]. On the basis of our results, it could be possible to suppose that 25HC might be involved in the progression of breast and ovarian tumors. In this regard, it should be taken into account that 25HC plasma levels increase after ingestion of a meal rich in oxysterols, as also detected after a dietary cholesterol challenge [25]. Indeed, increased cholesterol levels, which are often associated with obesity [71], are also detected in women with high risk of breast and ovarian cancer [72]–[74].

As it concerns the role of HIF-1α in apoptosis, its function remains somewhat controversial and appears to be cell type and context specific [75]–[78], a number of reports have demonstrated its contribution to programmed cell death during hypoxia [79]–[80]. Interestingly, it has been shown that hypoxia-induced HIF-1α activates ERα [81]–[82], further associating these transduction pathways to the intricate balance between factors that induce or counteract apoptosis in hypoxic conditions. To date, a large body of evidence has suggested that estrogen-activated ERα favorably influences cardiomyocyte survival both in vivo and in vitro model systems [8], [40], [83]–[85], explaining the gender-dependent protection against atherosclerosis and cardiovascular disease [86]. Our findings confirmed that E2 is able to prevent the hypoxia-induced apoptosis in cardiomyocytes and this ability was also exhibited by 25HC, which mimicked the potential of estrogen to regulate HIF-1α expression through ERα along with the rapid intracellular signaling such as ERK and p38 kinases.

Previous studies have demonstrated that HIF-1α is required for hypoxic induction of CTGF [47], which is involved in fibrotic processes in several tissues including the heart [21]. CTGF expression was found strongly up-regulated in atherosclerotic plaques [87]. Likewise, in vascular smooth muscle cells (VSMCs) CTGF overexpression induced apoptosis [88] and led to MMP2 activity [89] which would result in plaque destabilization. Accordingly, CTGF was mainly detected in complicated plaques where it stimulated mononuclear cell chemotaxis, acting as a pro-atherogenic factor [90]. An increased expression of CTGF was reported in infarcted and non-infarcted cardiac tissues [91]–[92], indicating CTGF as an attractive therapeutic target to treat fibrotic diseases and suggesting the use of its plasma levels as a biomarker for heart failure [21]. Indeed, our data have provided novel insight regarding the ERα-mediated ability of both E2 and 25HC to abolish the HIF-1α dependent up-regulation of CTGF in cardiomyocytes. Most importantly, 25HC displayed similar activity to E2 in preventing the hypoxia-induced expression of HIF-1α and CTGF in rat heart preparations, further extending to an in vivo model system its potential effects against hypoxic environments.

Here, we have demonstrated that 25HC activates the ER-mediated signaling in estrogen sensitive breast and ovarian cancer cells as well as in vascular model systems, hence mimicking the multifaceted estrogen action.

## Supporting Information

Figure S1 **mRNA expression of ERβ target genes.** (A) evaluation of SERPINF1, HSPA1L and SELENBP1 expression by real-time PCR in Hek293 cells transfected for 24 h with a vector or an ERβ expression plasmid. Data (mean ± SD) obtained from three independent experiments were normalized for 18S expression and shown as fold change of RNA expression upon treatment respect to cells treated with vehicle. (B) ERβ protein expression in Hek293 cells transfected with a vector or ERβ expression plasmid. β-actin serves as loading control.
(TIF)

Figure S2 **E2 and 25HC prevent CoCl2-induced apoptosis in HL-1 cells, as assessed by TUNEL staining.** Quantitative representation of data (mean ± SD) of three independent experiments. (°) indicates *p*<0.05 for HL-1 cells receiving vehicle (–) versus treatments, as indicated.
(TIF)

Figure S3 **Evaluation of HIF-1α and CTGF expression at both mRNA (A) and protein levels (B).** HL-1 cells were treated with 100nM actinomycin D (Act D) and exposed to hypoxia (2% O_2_) for 4h. (A) results obtained from experiments performed in triplicate were normalized for 18S expression and shown as fold change of RNA expression of cells exposed to hypoxia compared to cells cultured under normoxia. (B) immunoblots of HIF-1α and CTGF from HL-1 cells cultured under normoxia or hypoxia (2% O_2_), as indicated. β-tubulin serves as a loading control. (•), (°) indicate *p*<0.05 for cells cultured under normoxia versus cells exposed to hypoxia.
(TIF)

Figure S4 **25HC prevents the hypoxia-induced mRNA expression of HIF-1α and CTGF through ER and kinase-mediated signalling.** HIF-1α and CTGF mRNA expression evaluated by real-time PCR in HL-1 cells cultured under normoxia or hypoxia (2% O_2_) and treated with 10nM E2 and 1µM 25HC in combination with 10µM ICI, 10µM PD or 10µM SB, as indicated. Results obtained from experiments performed in triplicate were normalized for 18S expression and shown as fold change of RNA expression of cells exposed to hypoxia compared to cells cultured under normoxia. (•), (°) indicate *p*<0.05 for cells cultured under normoxia versus cells exposed to hypoxia.
(TIF)

Figure S5 **25HC (1µM) induces FAS mRNA expression in SkBr3 cells, as evaluated by real time PCR. Data (mean ± SD) obtained from three independent experiments were normalized for 18S expression and shown as fold change of RNA expression upon treatment respect to cells treated with vehicle.**
(TIF)
