# Supplementary figures and images for: The Cholesterol Metabolite 25-Hydroxycholesterol Activates Estrogen Receptor α-Mediated Signaling in Cancer Cells and in Cardiomyocytes
Source: PLoS One. 2011 Jan 31;6(1):e16631. doi: 10.1371/journal.pone.0016631 (PMC3031608; doi:10.1371/journal.pone.0016631)

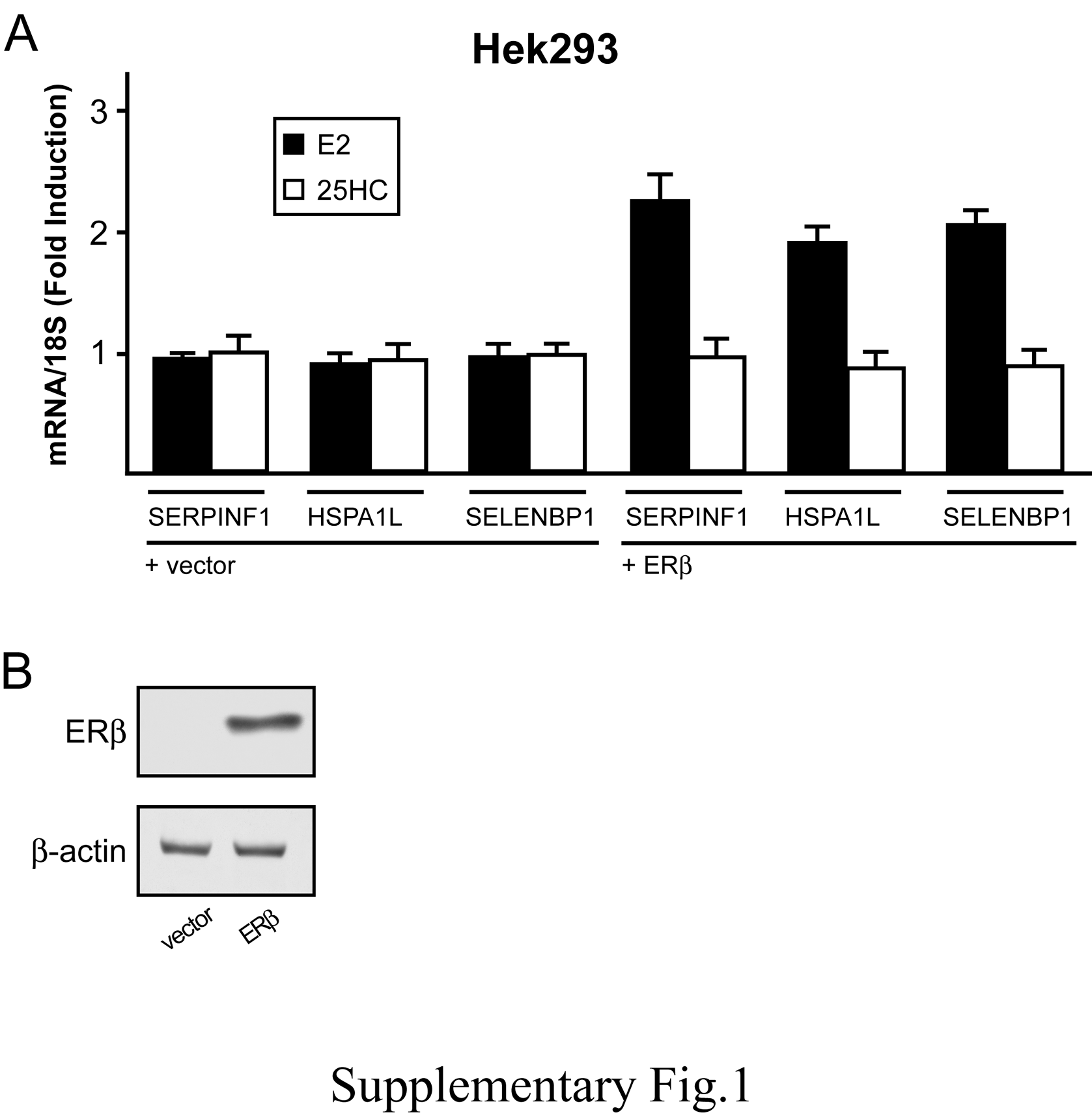

Supplement: Figure S1 — mRNA expression of ERβ target genes. (A) evaluation of SERPINF1, HSPA1L and SELENBP1 expression by real-time PCR in Hek293 cells transfected for 24 h with a vector or an ERβ expression plasmid. Data (mean ± SD) obtained from three independent experiments were normalized for 18S expression and shown as fold change of RNA expression upon treatment respect to cells treated with vehicle. (B) ERβ protein expression in Hek293 cells transfected with a vector or ERβ expression plasmid. β-actin serves as loading control. (TIF) [file pone.0016631.s001.tif]

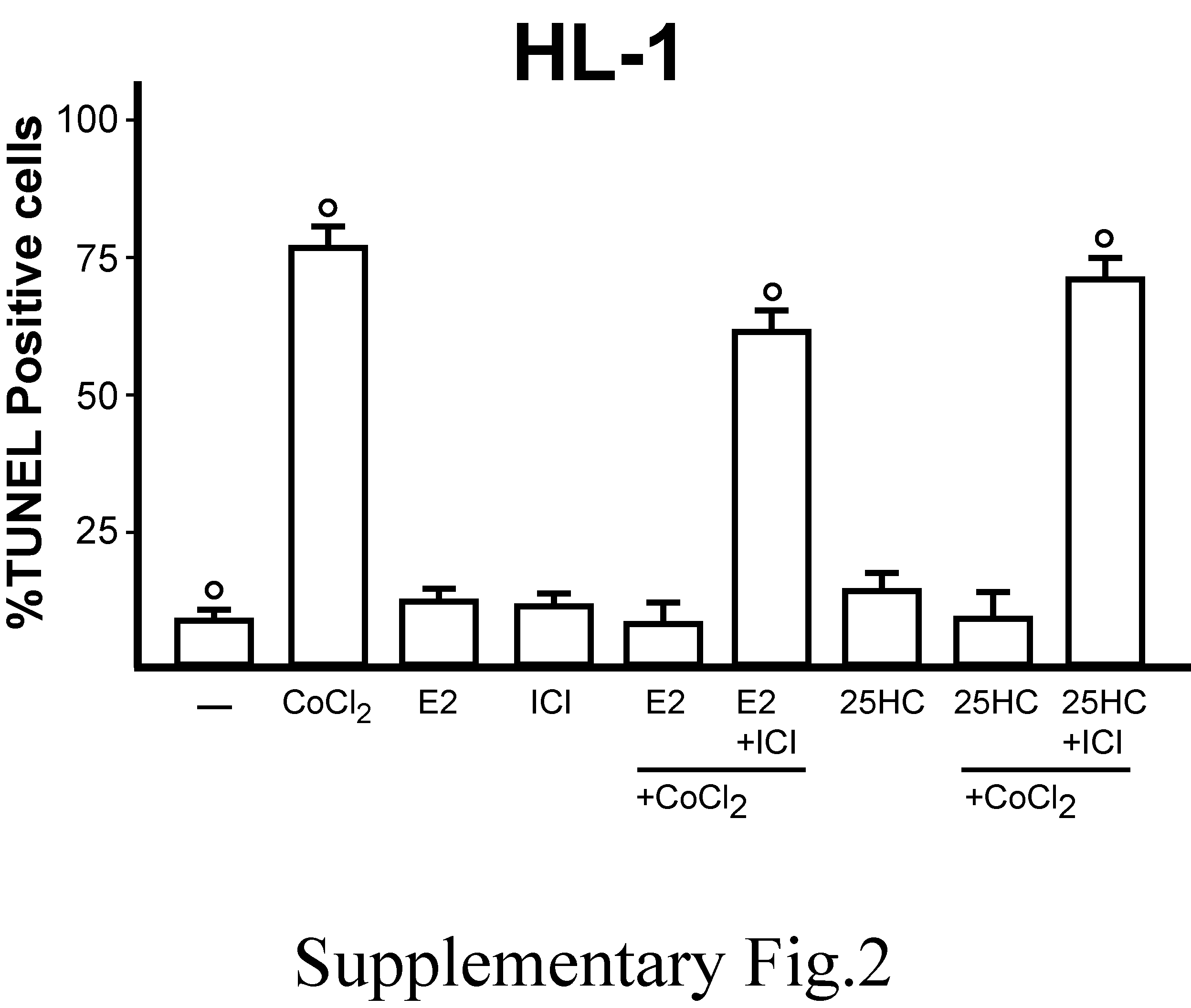

Supplement: Figure S2 — E2 and 25HC prevent CoCl2-induced apoptosis in HL-1 cells, as assessed by TUNEL staining. Quantitative representation of data (mean ± SD) of three independent experiments. (°) indicates p<0.05 for HL-1 cells receiving vehicle (–) versus treatments, as indicated. (TIF) [file pone.0016631.s002.tif]

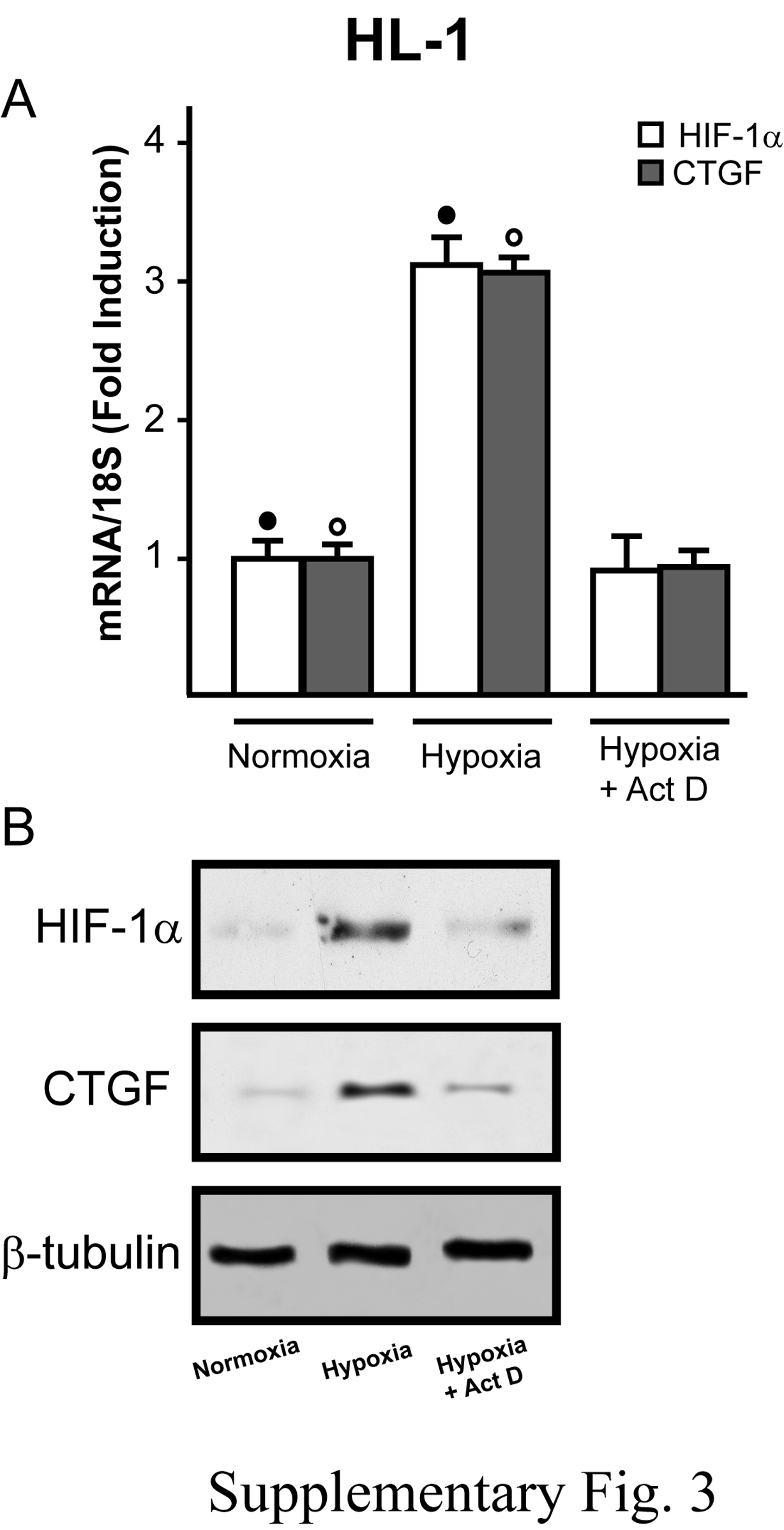

Supplement: Figure S3 — Evaluation of HIF-1α and CTGF expression at both mRNA (A) and protein levels (B). HL-1 cells were treated with 100nM actinomycin D (Act D) and exposed to hypoxia (2% O2) for 4h. (A) results obtained from experiments performed in triplicate were normalized for 18S expression and shown as fold change of RNA expression of cells exposed to hypoxia compared to cells cultured under normoxia. (B) immunoblots of HIF-1α and CTGF from HL-1 cells cultured under normoxia or hypoxia (2% O2), as indicated. β-tubulin serves as a loading control. (•), (°) indicate p<0.05 for cells cultured under normoxia versus cells exposed to hypoxia. (TIF) [file pone.0016631.s003.tif]

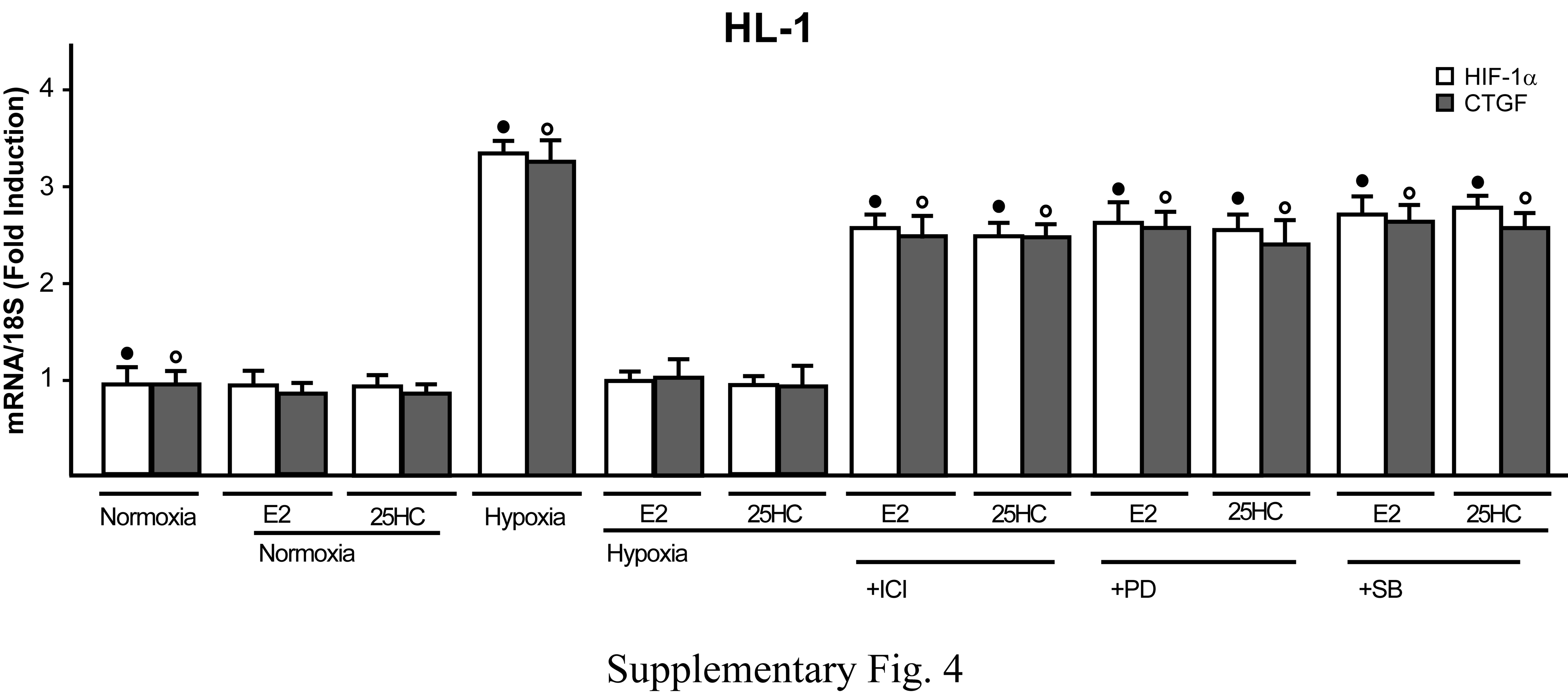

Supplement: Figure S4 — 25HC prevents the hypoxia-induced mRNA expression of HIF-1α and CTGF through ER and kinase-mediated signalling. HIF-1α and CTGF mRNA expression evaluated by real-time PCR in HL-1 cells cultured under normoxia or hypoxia (2% O2) and treated with 10nM E2 and 1µM 25HC in combination with 10µM ICI, 10µM PD or 10µM SB, as indicated. Results obtained from experiments performed in triplicate were normalized for 18S expression and shown as fold change of RNA expression of cells exposed to hypoxia compared to cells cultured under normoxia. (•), (°) indicate p<0.05 for cells cultured under normoxia versus cells exposed to hypoxia. (TIF) [file pone.0016631.s004.tif]

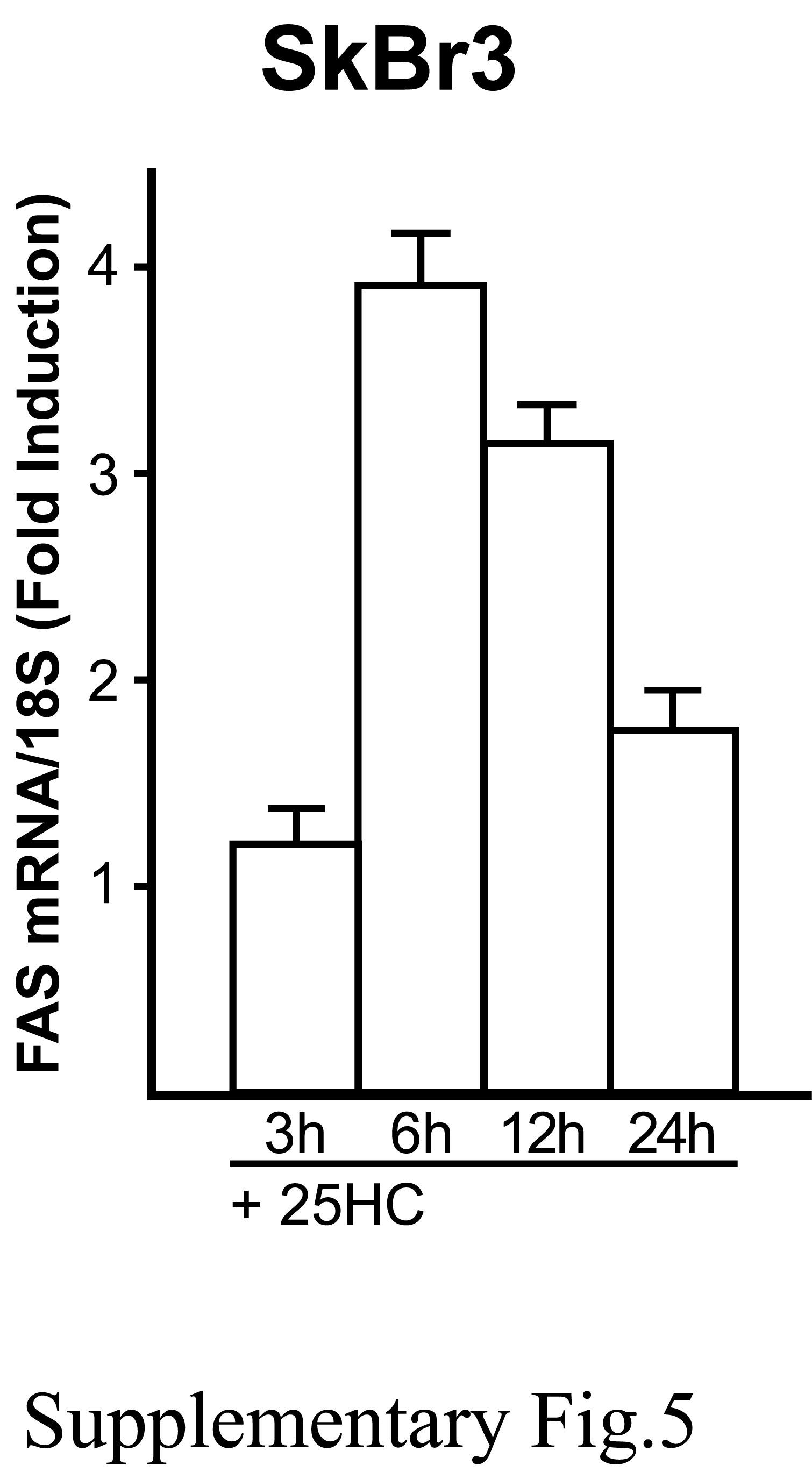

Supplement: Figure S5 — 25HC (1µM) induces FAS mRNA expression in SkBr3 cells, as evaluated by real time PCR. Data (mean ± SD) obtained from three independent experiments were normalized for 18S expression and shown as fold change of RNA expression upon treatment respect to cells treated with vehicle. (TIF) [file pone.0016631.s005.tif]
